# CYP19A1 promotes gastric cancer as part of a lipid metabolism-related gene signature related to the response of immunotherapy and prognosis

**DOI:** 10.1186/s12920-023-01664-y

**Published:** 2023-10-02

**Authors:** Xinyi Zhou, Fanyu Meng, Linmei Xiao, Hua Shen

**Affiliations:** 1 Nanjing BenQ Medical Center, The Affiliated BenQ Hospital of Nanjing Medical University, NanjingJiangsu Province, 210019 China; 2 Department of General Surgery, the First Affiliated Hospital of Nanjing Medical University, 300 Guangzhou Road, Nanjing, 210029 Jiangsu China

**Keywords:** CYP19A1, Gastric cancer, Lipid metabolism, Immunotherapy

## Abstract

**Background:**

Increasing evidence suggests that the metabolism of lipids plays a crucial role in the progression of gastric cancer. However, the expression of lipid metabolism-related genes (LMGs) still does not serve as a prognostic biomarker in gastric cancer.

**Methods:**

We obtained transcriptome data for 751 LMGs and divided STAD patients into two subtypes based on differences in LMGs expression. Then, we analyzed genetic changes in two subtypes as well as immune features to determine their differences. We also constructed a prognostic risk model related to LMGs for individualized comprehensive evaluations.

**Results:**

In this study, two lipid metabolic (LM) subtypes were identified anchored in the expression profiles of LMGs. Clinical information, genomic alterations, immune features, and immunotherapy response varied significantly between the two LM subtypes. A risk model based on LMGs was also developed to assess prognosis and distinguish patients with high risk from those at low risk. The prognosis differed significantly between the two risk groups of patients. In STAD patients, the risk score was strongly correlated with genomic alterations and immune profile scores. Also, the risk score was an excellent predictor of immune checkpoint inhibitors (ICIs) response. Anchored in preliminary results derived from the aforementioned bioinformatic analysis, we chose CYP19A1 as our target gene and the expression of CYP19A1 was verified in several common gastric cancer cell lines. Then, we carried out the Western blotting, CCK-8 assay, colony formation assay, wound healing assay, and transwell assay to explore the effects of CYP19A1 on malignant biological behavior, and positive consequences were obtained.

**Conclusions:**

In this study, STAD patients were divided into two subtypes based on LMGs expression. It is possible to assess the prognosis of a patient and the response to immunotherapy using the established prognostic risk model. A series of basic laboratory experiments also verified the functional role of CYP19A1 in gastric cancer.

**Supplementary Information:**

The online version contains supplementary material available at 10.1186/s12920-023-01664-y.

## Introduction

Gastric cancer (GC) is a prevalent malignant tumor and the third leading cause of cancer-related deaths globally [1]. The better Lauren/WHO classification and tumor-node-metastasis (TNM) staging system of gastric cancer subtypes have enabled recent advances in treatment [2]. Some patients can improve their survival rate through surgery, chemotherapy, radiotherapy, or targeted therapies. These treatments, however, have limited effects on different patients because of tumor heterogeneity and difficulty in early diagnosis [3, 4]. The 5-year survival rate of patients diagnosed with GC is around 30%, and yet 70% of them do not benefit from these treatments [5]. In order to establish a new predictive and diagnostic tool, we must explore novel biomarkers.

A significant characteristic of cancer cells is their lipid metabolism [6]. Maintenance of homeostasis and cell function is dependent on lipids [7]. As a component of the cell membrane, lipids have crucial effects on cell growth and the different pathways the cell follows [8]. The metabolism of lipids is reprogrammed by tumor cells to meet the increased demand for lipids [9]. Reprogrammed lipid metabolism is present in STAD, providing energy storage and intermediates for a variety of metabolic activities involved in tumor cell proliferation and metastasis [10]. According to in-depth studies about tumor mutation burden, large numbers of potential prognostic markers for immunotherapy in malignant tumors have surfaced [11, 12], Among many biological processes, the association between lipid metabolism and immunotherapy has become a research hotspot [13, 14]. The metabolic state and functional phenotypes of cancer cells and tumor-infiltrating immune cells could be affected by metabolic adaption and nutrient competition for essential nutrients. In addition, reprogramming lipid metabolism may enhance tumor immunotherapy by disrupting effector T cell senescence. A cancer patient's survival and response to immunotherapy can be predicted by a gene signature related to lipid metabolism [15, 16]. Further studies remain performed to determine whether the expression of LMGs could serve as a biomarker of prognosis and immunotherapy response in patients with STAD.

To identify hub genes that could predict patients’ survival, we investigated LMGs expression in STAD. A 5-gene prognostic signature was developed and validated to accurately predict STAD patients' prognosis and immunotherapy response. Then, we conducted relevant experiments to demonstrate the function of the most significant gene of the aforementioned 5-gene prognostic signature. Identifying new therapeutic targets for STAD will be made easier with this prognostic signature.

## Methods

### Datasets

We downloaded normalized transcriptome data of 373 samples (343 tumor samples and 30 normal samples) and clinical information from The Cancer Genome Atlas (TCGA) (https://tcga-data.nci.nih.gov/tcga). Patients with missing survival data or with overall survival (OS) of less than 30 days were excluded, leaving 317 patients for further analysis. Gene expression data was also accessed from the Genotype-Tissue Expression (GTEx) database (https://gtexportal.org/). The STAD external validation cohort (including 433 samples, GSE84437) was downloaded from the GEO database (https://www.ncbi.nlm.nih.gov/geo/). The "limma" R package was used to normalize the data on RNA expression in this dataset.

### The identification of STAD groups

The 751 LMGs were used for differential expression analysis (*P* < 0.05, |log2FC|> 1.5) and univariate Cox regression analysis, and intersecting LMGs were found. In our study, we used the “ConsensusClusterPlus” R package to categorize STAD patients anchored in the expression patterns of prognostic LMGs-related differentially expressed genes (DEGs). A suitable clustering number could be determined using cophenetic, dispersion, and silhouette indicators. The t-distributed stochastic neighbor embedding (tSNE) algorithm was used to validate clustering results. Then we showed the expression levels of prognostic LMGs-related DEGs of two subtypes. The Kaplan–Meier (K-M) curves were displayed to compare the overall survival of two subtypes. The clinical data (status, stage, M stage, N stage, T stage) of STAD patients in the TCGA cohort were collected and analyzed in combination with subtypes. In order to investigate functional annotations and pathways between two subtypes, the Gene Ontology (GO), Kyoto Encyclopedia of Genes and Genomes (KEGG) [17–19], and GSVA analyses were conducted.

### Analysis of genomic characteristics

We downloaded mutation annotation format (MAF) profiles of TCGA STAD patients from the TCGA data portal (https://portal.gdc.cancer.gov/). The Maftools R package was used to analyze STAD mutation data, and a tumor mutation burden (TMB) score was calculated for every STAD patient. Patients with high and low TMB levels were compared in terms of survival. Furthermore, the copy number alteration (CNA) data of STAD patients were obtained from the TCGA database. We used GISTIC 2.0 to determine whole genome amplifications and deletions. We calculated the CNA burden at the focal and arm levels between two subtypes.

### Analyzing the immune microenvironment and the response to immunotherapy

Using Single-sample gene set enrichment analysis (ssGSEA), we quantified the scores of 21 immune cells and 21 immune functions. We used the CIBERSORT algorithm with 1,000 permutations to quantify the compositions of 22 immune cell types. As ICIs are commonly used in cancer treatment, we also calculated the scores of 50 immune checkpoints using the ssGSEA algorithm. The immune scores, stromal scores, and ESTIMATE scores of STAD patients were quantified using the ESTIMATE algorithm. Considering the important role of immunotherapy in cancer treatment, we evaluate the potential immunotherapy responses between two subtypes with the Tumor Immune Dysfunction and Exclusion (TIDE) algorithm online (http://tide.dfci.harvard.edu/). We then used subclass mapping algorithms to identify PD-1, PD-L1, and CTLA4 therapeutic effects in two subtypes of patients. Through the R package 'oncoPredict', we assessed drug sensitivity in two subtypes and identified potential targeted therapy drugs.

### The construction of a prognostic signature linked to LMGs

The prognostic-related LMGs were used in the random forest algorithm to identify the hub genes. The median risk score was evaluated to divide patients into high- and low-risk groups making use of multivariate Cox regression analysis. In the TCGA cohort, the K-M survival curve was used to estimate the prognosis for patients in both risk groups based on overall survival, progression-free survival, and disease-free survival. A K-M survival curve was also used to validate the prognostic value of LMGs in GC patients in the GEO cohort. A receiver operating characteristic (ROC) curve analysis was then used to evaluate the accuracy of LMGs in predicting STAD survival probability. We compared the differences in the expression of hub genes in normal and tumor tissues and used the expression of hub genes to make survival curves.

### A comparison of TMB levels and stemness index

For each patient, we showed the distribution of risk scores and clinical features. A correlation was then shown between the risk score and the TMB levels. By using the OCLR algorithm, we quantified the DNA methylation-based stemness index (mDNAsi) and the mRNA expression-based stemness index (mRNAsi). Furthermore, we analyzed the difference between the two groups in mDNAsi and mRNAsi.

### Analysis of immune terms

SsGSEA was used to compare scores between two groups for 21 immune cells, 21 immune functions, and 50 immune checkpoints. Additionally, we examined the correlation between risk scores and immune cell scores (measured by ssGSEA and CIBERSORT). We then compared immune scores, stromal scores, and ESTIMATE scores between two groups of STAD patients. We also investigated whether high-risk versus low-risk patients respond differently to immunotherapy with TIDE and subclass mapping analysis. To confirm our prediction, we compared the immunotherapy response between two groups of STAD patients in the TCGA.

### Cell culture, RNA extraction, and quantitative real-time PCR

We obtained several human GC cell lines from the cell bank of the Chinese Academy of Sciences (Shanghai, China) and employed them in this research cultured in RPMI 1640 medium (Invitrogen) supplemented with regular 10% fetal bovine serum (FBS, WISENT, Canada) as well as 1% antibiotics (100 U/ml penicillin and 100 mg/ml streptomycin) in a humidified atmosphere containing 5% carbon dioxide at 37 °C. We extracted RNA from cells making use of Trizol Reagent (Invitrogen, Carlsbad, CA, USA). After reverse transcription, we measured the expression of mRNA by quantitative real-time PCR (ABI 7300) with SYBR Green assay (Vazyme Biotech Co., Ltd, Nanjing, China), and the data was calculated by the 2 − ΔΔCT method. The primers used are as shown below: CYP19A1 forward, 5′-CACCCATCTTTGCCAGGTAGTC-3′ and CYP19A1 reverse, 5′-ACCCACAGGAGGTAAGCCTATAAA-3′; GAPDH forward, 5′-TGCACCACCAAC TGCTTAGC-3′ and GAPDH reverse, 5′-GGCA TGGACTGTGGTCATGAG-3′. Experiments were performed in triplicate.

### Plasmids construction, siRNA interference, and transfection

CYP19A1-overexpressing plasmids were constructed into the pGL3-basic vector (Promega, Madison, WI, USA) and the siRNA sequences designed against CYP19A1 are listed as follows: CYP19A1-1: CUUUGGGAAUAAUAAUCGUUCAGGA, CYP19A1-2: UCCUGAACGAUUAU UAUUCCCAAAG, NC: UUCUCCGAACGUGUCACGUTT.

### Western blotting

We extracted total protein from selected cell lines using RIPA lysis buffer (Beyotime, Shanghai, China), separated isolated proteins through sodium dodecyl SDS-PAGE and transferred them to a PVDF membrane. Then, 5% non-fat milk was used to block the membranes at room temperature for 1.5 h and we incubated the membranes with the specific primary antibodies at 4 °C overnight. We used TBST to wash the membranes before and after the incubation with secondary antibodies. We employed an ECL detection system to detect the relative expression levels of the proteins. The listed antibodies were used: CYP19A1 (Abcam, Britain); N-cadherin, E-cadherin, Vimentin, and GAPDH (Proteintech, Wuhan, China). The membrane was cut during the process of western blots according to the molecular mass of the target protein prior to hybridization with antibodies in order to save the use of PVDF membrane, which resulted in the absence of images of full-length blots. The unedited images of blots in Fig. 10 and their replicates are accessible in the Additional file 1.

### CCK-8 assay

Cell Counting Kit-8 (CCK-8) (Beyotime, Shanghai, China) was used to evaluate cell proliferation according to the manufacturer’s recommendations. We plated the cells in 96-well plates (1000 cells/well) containing RPMI 1640 supplemented with 10% FBS for 5 days. During this assay, we added 10 μl of CCK-8 reagent to each well and incubated the cells at 37 °C for 2 h. The spectrophotometer was used to determine the absorbance at 450 nm. Each group was evaluated in triplicate.

### Colony formation assay

500 MKN45 or AGS cells treated with siRNA or plasmids were plated in a six-well plate and cultured in RMPI-1640 medium containing 10% FBS for 14 days. Methanol was used to fix proliferating colonies and we made use of 1% crystal violet (Beyotime, Shanghai, China) to stain proliferating colonies. The colonies were photographed and then counted three times.

### Wound healing assay and transwell assay

For the wound healing assay, we seeded cells in 6-well plates and cultured them to the subfusion state. Then, a 200 μl sterile pipette tip was used to create linear scratch wounds. We captured images at both 0 and 48 h and evaluated cell healing rates. For the transwell assay, we used 6.5 mm chambers with 8 μm pores (Corning Costar Corp., USA) to evaluate the migratory and invasive abilities of gastric cancer cells. In this experiment, we plated 2 × 10^4^ cells in the upper chamber cultured in 200 μl of serum-free RMPI-1640 medium, and 600 μl of the RMPI-1640 medium containing 10% FBS without cells was added to the lower chamber. After incubating for 48 h, we stained the cells having migrated to the lower surface of the filter with 1% crystal violet for 30 min. For invasion assays, we added 0.1 ml of Matrigel (50 μg/ml, BD Biosciences, USA) onto the upper chamber. Then, the cells were plated, and the other procedures were similar to the aforementioned steps. The experiments were performed in triplicate.

### Statistical analysis

All statistical analyses were performed using R software (version 4.2.1). In the analysis of statistical significance between the two groups, a Student t-test was used. We estimated the correlation between two parameters using Spearman's correlation analysis. Multivariate cox regression was used to determine the model and the random forest algorithm was applied to further identify prognostic genes. mRNA expression levels and the number of colonies were tested by Student’s t test. Two-tailed *p* < 0.05 was defined as statistically different.

## Results

### Identification of lipid metabolic-related subtypes

**Fig. 1:**
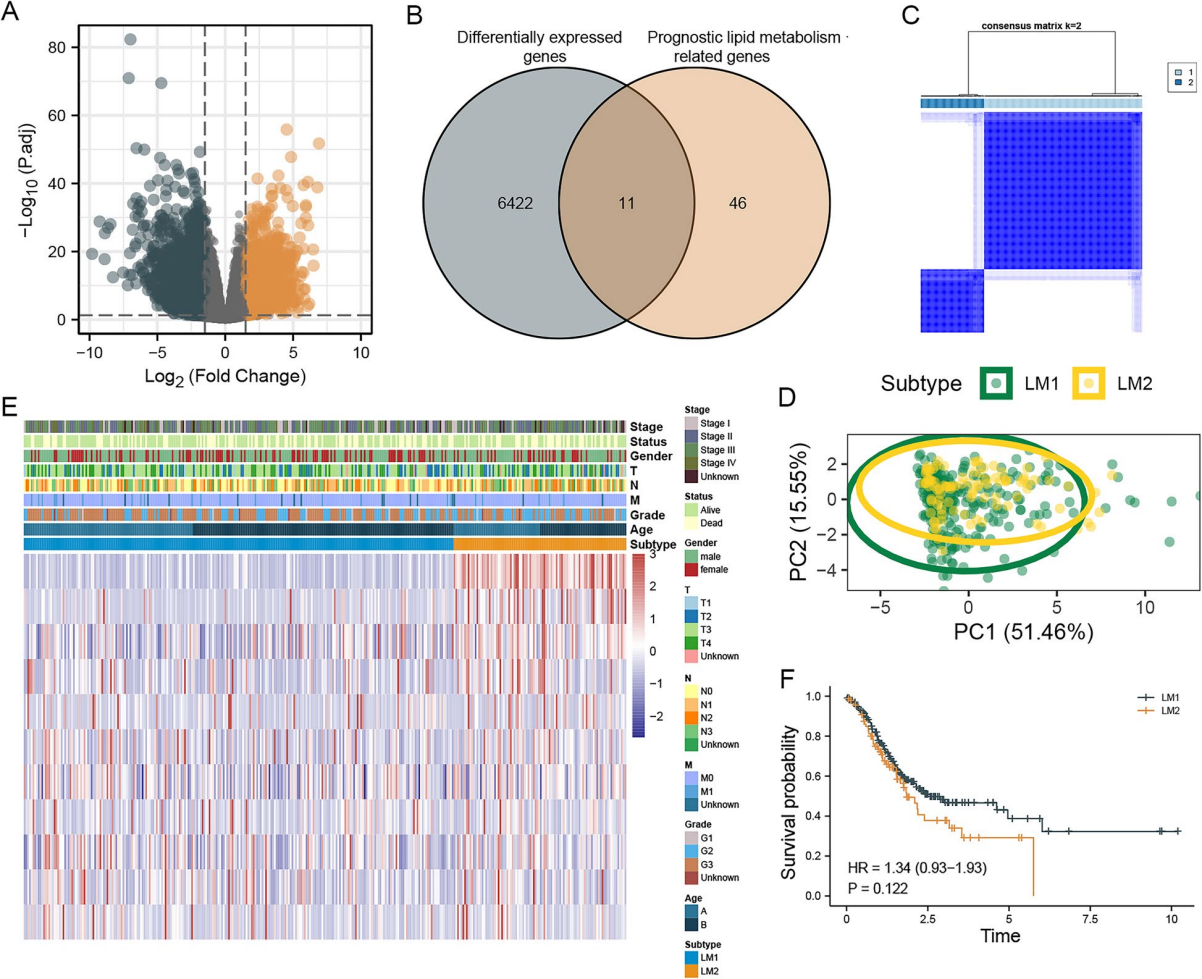
Identification of lipid metabolism-related subtypes. Notes: **A** The 6433 differentially expressed genes were presented. **B** The 11 differentially expressed genes associated with lipid metabolism and prognosis were identified. **C** Different clusters of the aforementioned 11 genes cohort were identified for k = 2. **D** The differences in distribution between LM1 and LM2 in the tSNE were analyzed. **E** The distribution of clinical characteristics and the expression levels of LMGs were displayed in a heatmap. **F** The survival curve between LM1 and LM2 was drawn

By comparing the difference in gene expression between normal and tumor tissue, 6433 different genes were identified (Fig. 1A). Univariate Cox regression analysis screened out 57 LMGs associated with prognosis. The intersection between differential express genes and prognosis-related LMGs contained eleven genes (Fig. 1B). The consensus clustering analysis revealed that the correlations were strong between two clusters when k = 2, which indicated that the 343 STAD patients could be well divided into two subtypes based on the eleven LMGs, namely LM1 (*n* = 245) and LM2 (*n* = 98) (Fig. 1C). There were great differences in distribution between the two subtypes in the tSNE (Fig. 1D). In the heatmap, eleven LMGs were shown with their different expression levels (Fig. 1E). Survival curves between the two subtypes showed a tendency to differ. There was a lower survival rate among patients in LM2 compared with LM1 (Fig. 1F).

**Fig. 2:**
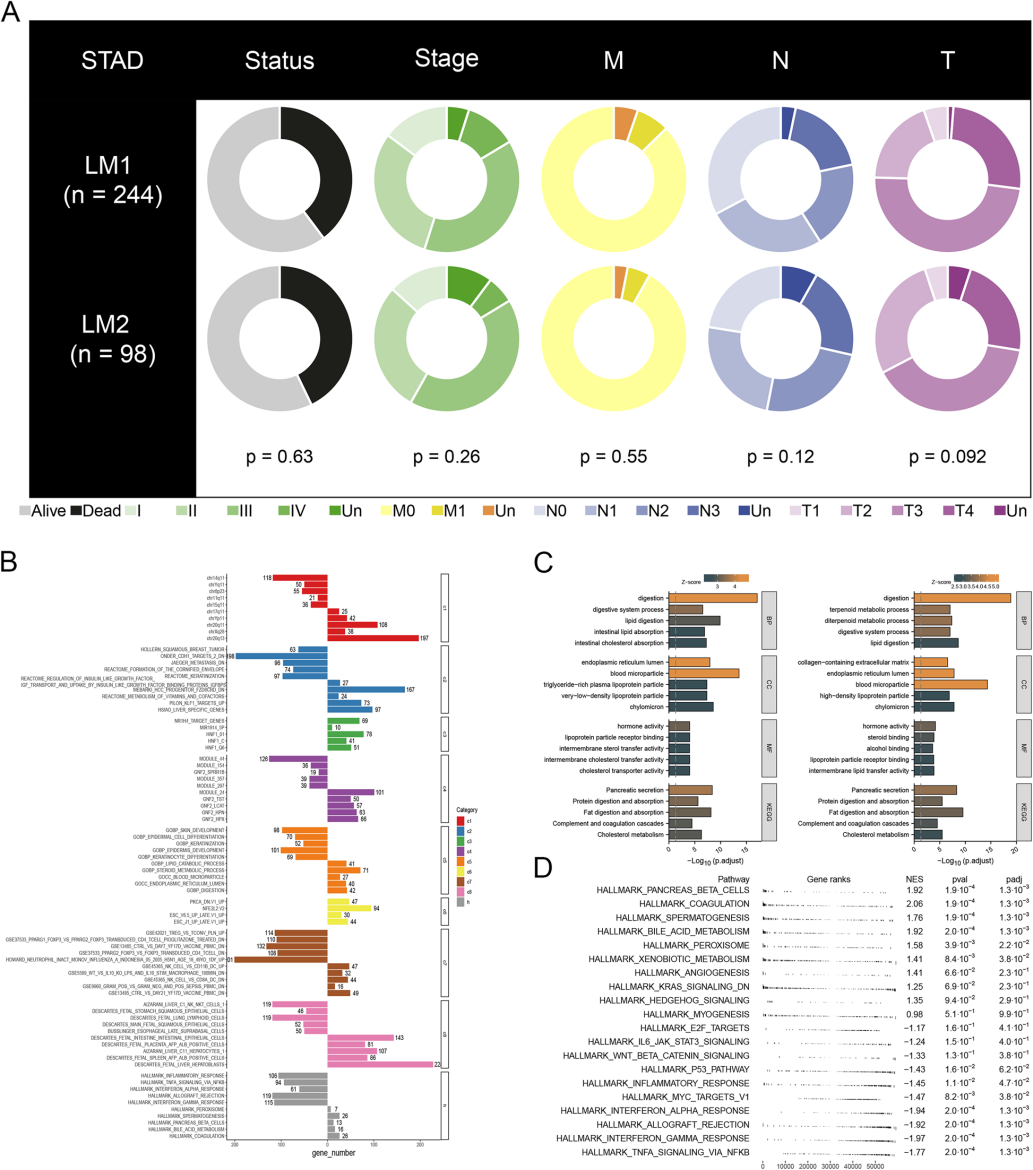
Function prediction of two subtypes. Notes: **A** The differences in clinicopathologic features between two subtypes of gastric cancer examined by the Chi-squared test were presented in pie charts. **B** GSEA analysis was performed to predict the pathways and functions associated with LM1 and LM2. **C** The top 20 GO and KEGG signaling pathways in the two subtypes were shown. **D** The top 20 Hallmark signaling pathways in two subtypes were shown

Then we compared the clinical features of STAD patients in two subtypes and there were no significant differences (Fig. 2A). In order to determine the pathways and functions of LM1 and LM2 enrichment, we used nine gene sets for GSEA analysis (Fig. 2B). There was a stronger correlation between LM1 and lipid-related pathways, such as intestinal lipid absorption, than LM2 (Fig. 2C). In addition, the top five markedly enriched pathways in LM1 included pancreas beta cells, coagulation, spermatogenesis, bile acid metabolism, and peroxisome. LM2 showed great enrichment in five pathways: E2F targets, IL-6 JAK STAT3 signaling, WNT beta-catenin signaling, P53 pathway, and inflammatory response (Fig. 2D).

### Analysis of genomic alterations between two subtypes

**Fig. 3:**
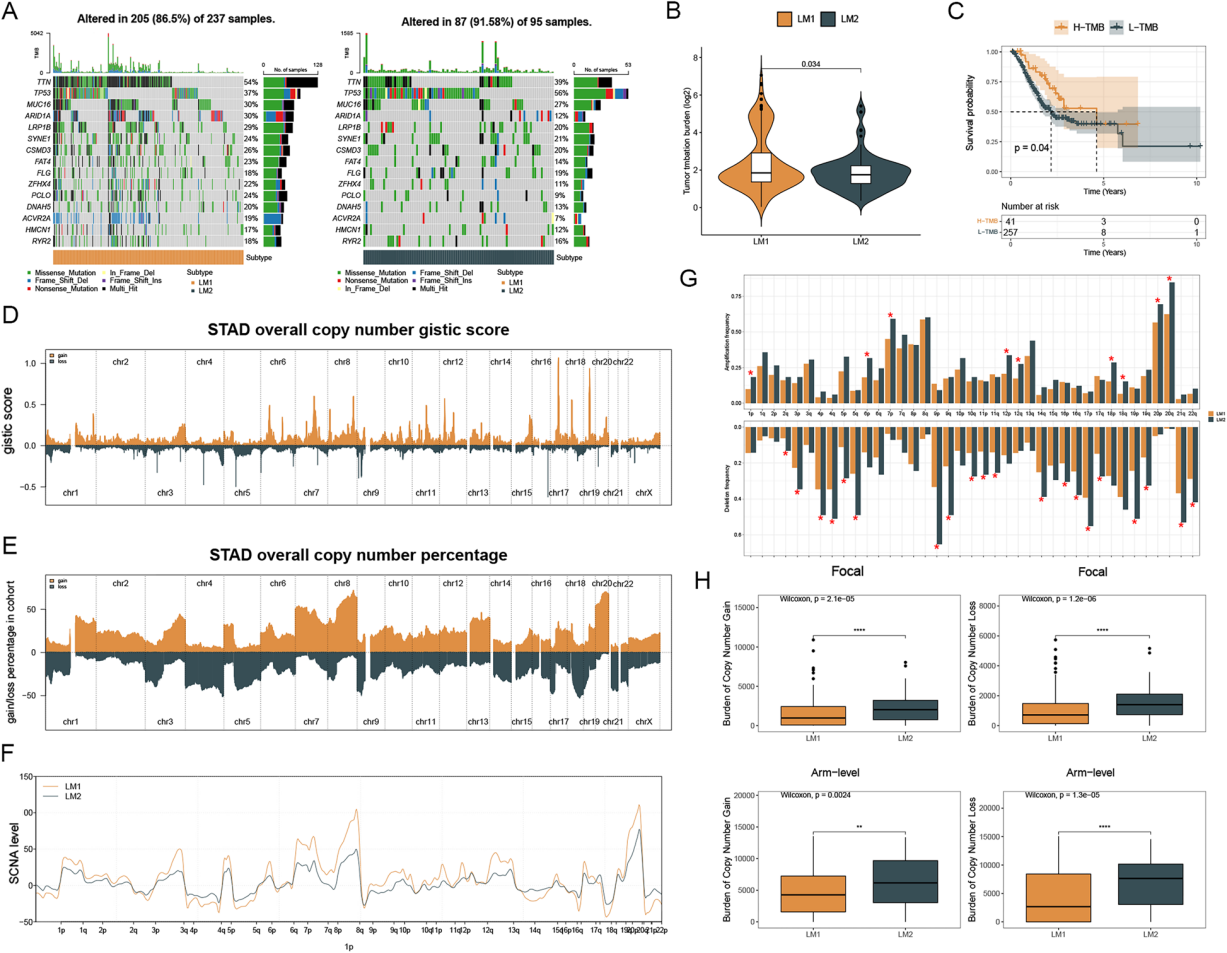
Analysis of genomic alterations between two subtypes. Notes: **A** Mutation profiles of two subtypes were presented. **B** The TMB level of patients in both two subtypes was calculated. **C** The survival rate of patients with different TMB levels was analyzed. **D** The gistic score of copy number variation of TCGA-STAD was shown. **E** The gain and loss percentage of copy number variation of TCGA-STAD. **F** The somatic copy-number alterations (SCNA) levels in two subtypes. **G** The frequency of autosomal amplification and deletion between patients in LM1 and LM2 was analyzed. **H** The burden of gain and loss of two subtypes both at the focal and arm levels was shown

In order to investigate the difference in gene mutations between LM1 and LM2, we calculated genetic mutation levels. Based on the somatic mutation profiles, patients in LM1 had specific top mutated genes and a higher TMB level than those in LM2 (Fig. 3A, B). There was a better survival rate for patients with high TMB levels than for patients with low TMB levels, as revealed by the survival curve (Fig. 3C). Gistic scores and percent distributions in two subtypes were shown, and a line diagram was used to visualize the differences in somatic copy number alternations (SCNAs) between the two subtypes (Fig. 3D-F). It was found that the frequency of autosomal amplification and deletion was significantly different between patients in LM1 and LM2 (Fig. 3G). LM1 patients had a lower burden of gain and loss, both at the focal and arm levels than LM2 patients (Fig. 3H).

### Immunity and immunotherapeutic responses in two subtypes

**Fig. 4:**
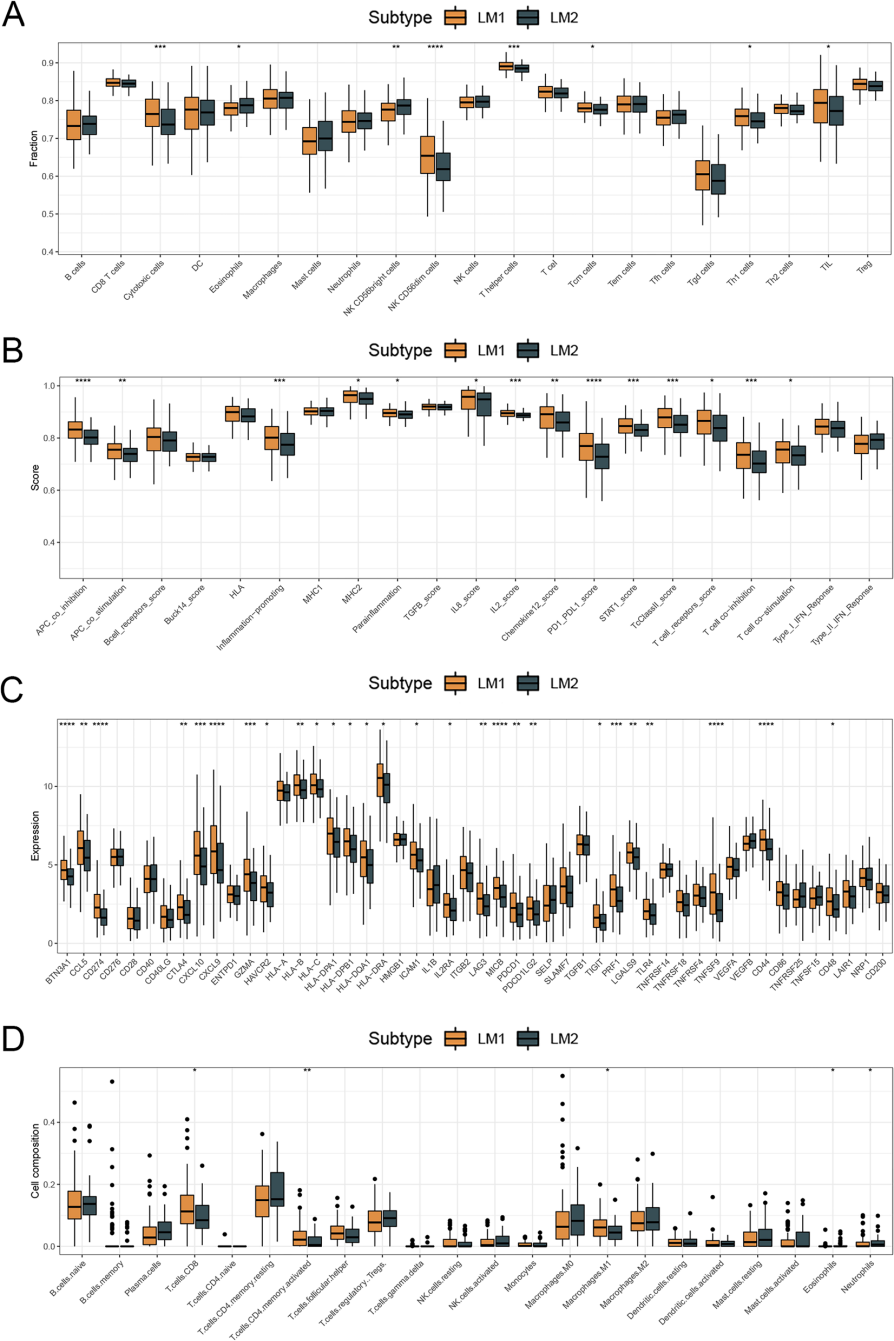
Differences in immunity between two subtypes. Note: **A** and **B** The immune cells and immune functions of two subtypes were estimated. **C** and **D** The expression level of immune checkpoints and immune checkpoints’ scores were compared between patients in LM1 and LM2

**Fig. 5:**
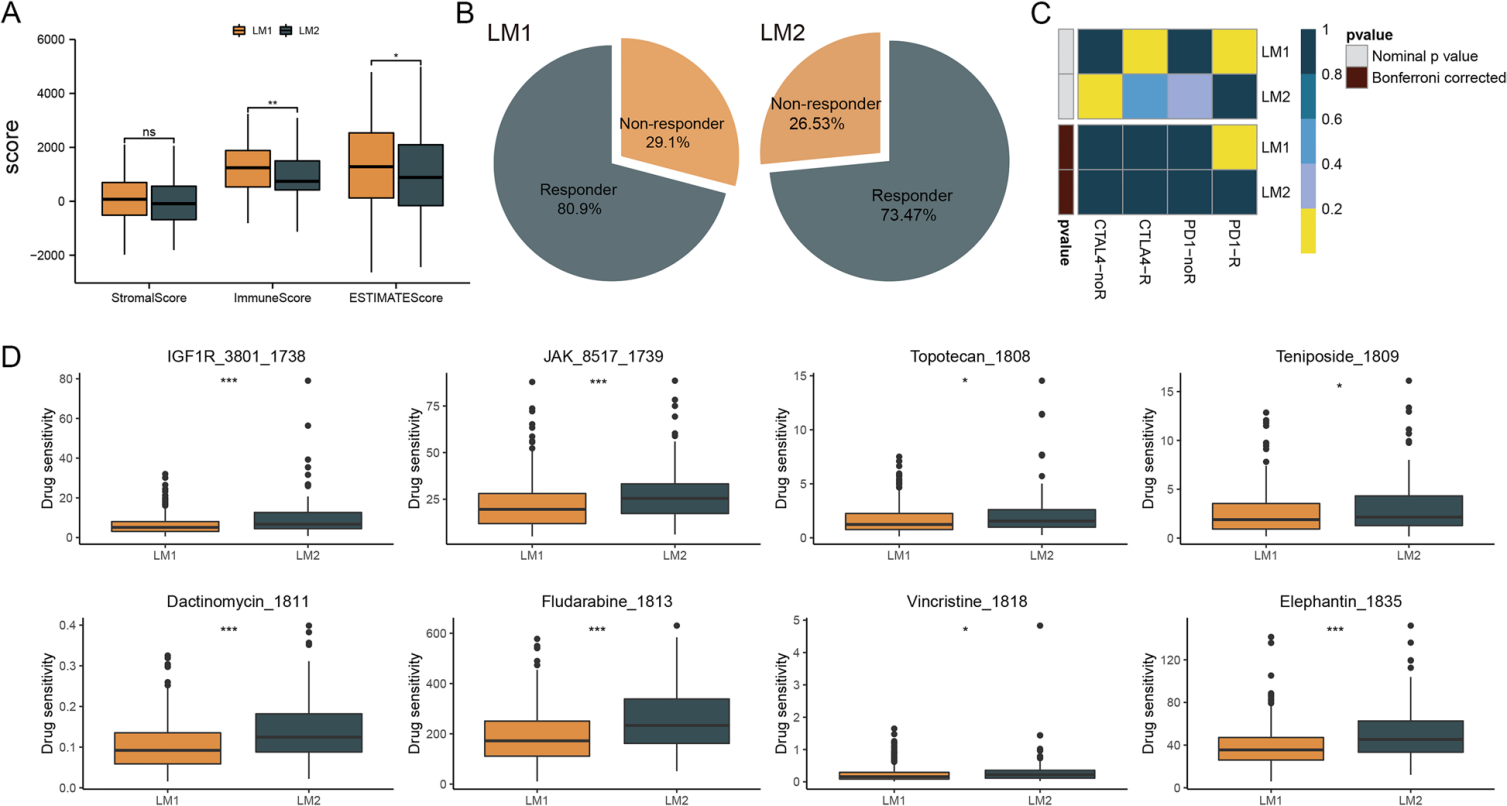
Immunotherapeutic responses in two subtypes. Notes: **A** The immune, stromal, and ESTIMATE scores were compared between two subtypes. **B** Patients in LM2 have a lower percentage of responders. **C** Subclass mapping analysis showed differences in response to PD1 treatment between patients in LM1 and LM2. **D** The predicted IC50 for 8 common chemo drugs was presented

It has become increasingly clear that LMGs are responsible for modulating the sensitivity of cancer cells to immunotherapy [20]. The ssGSEA algorithm was used to compare scores of immune cells between two subtypes, and there was a significant difference between them (Fig. 4A). LM1 patients had higher immune function scores than LM2 patients (Fig. 4B). The tumor immune microenvironment was also analyzed using the CIBERSORT algorithm. There was evidence that CD8 + T cells play a major role in anti-tumor activity [21], and patients in LM1 had higher CD8 + T cell scores than patients in LM2 (Fig. 4C). In addition, we compared immune checkpoints’ scores between patients in LM1 and LM2 and found that most of the patients in LM1 had higher immune checkpoints’ scores than those in LM2 (Fig. 4D). According to the ESTIMATE analysis, patients in LM1 had higher immune, stromal, and ESTIMATE scores than those in LM2 (Fig. 5A). As a result, we compared the immunotherapeutic response between two subtypes. TIDE results showed that patients in LM1 responded better to immunotherapy than those in LM2 (Fig. 5B). Subclass mapping analysis showed differences in response to PD1 treatment between two subtypes of patients (Fig. 5C). For both groups of patients, we presented eight drugs that may be useful (Fig. 5D).

### Identification of prognostic hub genes

**Fig. 6:**
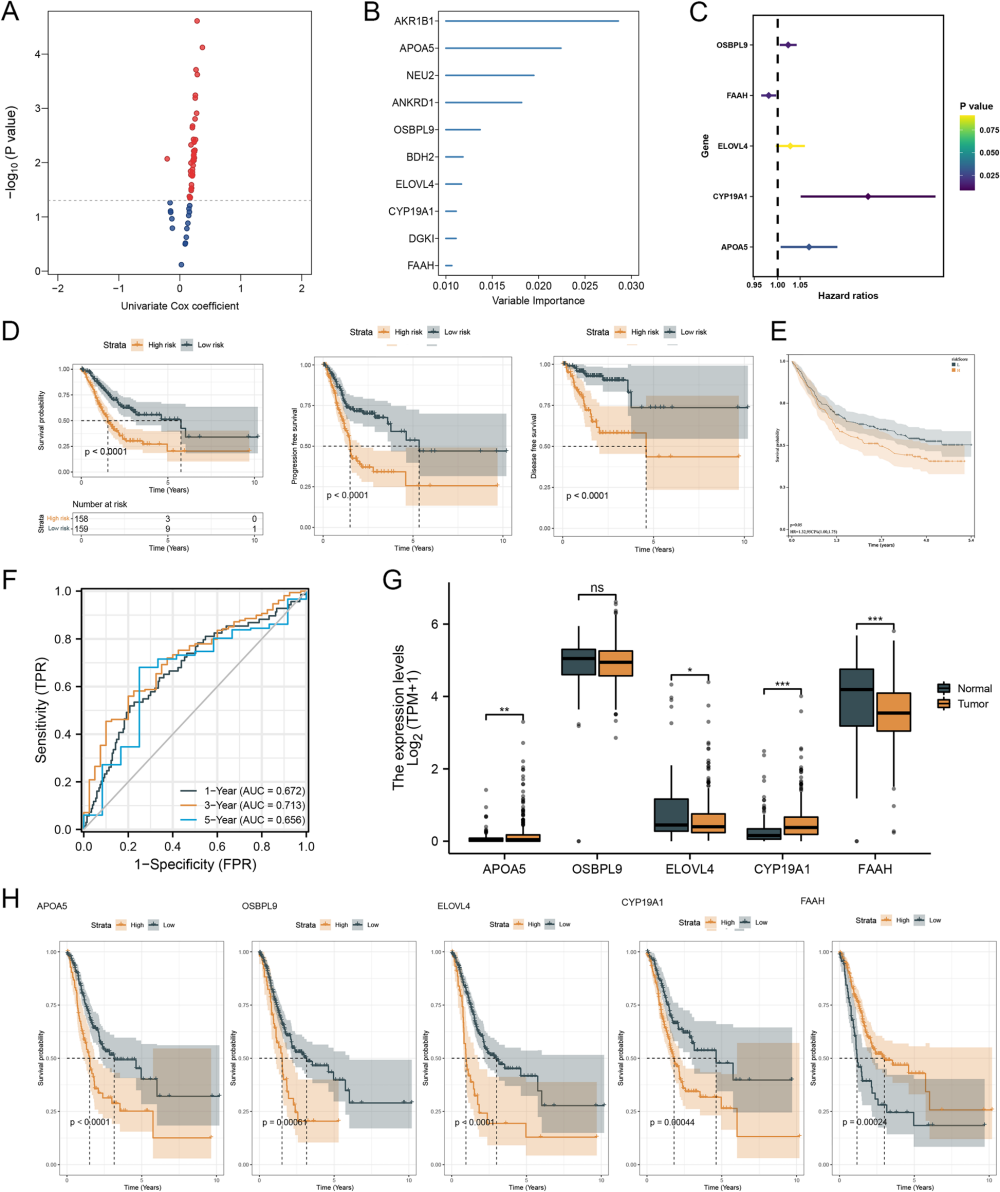
Identification of prognostic hub genes. Notes: **A** and **B** The hub genes related to LMGs were screened by random forest analysis. **C** The survival analysis of 5 hub genes related to LMGs was performed. **D** OS, PFS, and DFS in the TCGA-STAD cohort were estimated by Kaplan-Meier curves in the two groups. **E** OS in GSE84437 was shown by Kaplan-Meier curves. **F** The accuracy of the expression of LMGs in predicting OS was identified by ROC curves. **G** The expression levels of these five hub genes were compared between tumor and normal tissues. **H** The survival analysis anchored in the expression level of the five hub genes was carried out

To verify the hub genes related LMGs, random forest analysis was performed (Fig. 6A, B). A total of five hub genes related to LMGs were identified, which were subsequently analyzed. For survival outcomes, four genes were risky and one was protective (Fig. 6C). According to the K-M survival curves, patients with high hazard had worse overall survival, progression-free survival, and disease-free survival than those in the low-risk group in TCGA cohorts (Fig. 6D). Then we validated the same result with the GEO cohort (Fig. 6E). In addition, we used ROC curves to identify the accuracy of the expression of LMGs in predicting 1-, 3- and 5-year overall survival (Fig. 6F). The expression of these five hub genes was compared between normal and tumor tissues (Fig. 6G). K-M survival curves based on five gene expression were shown (Fig. 6H).

### Correlation of LMGs with clinical features, TMB levels, and stemness index

**Fig. 7:**
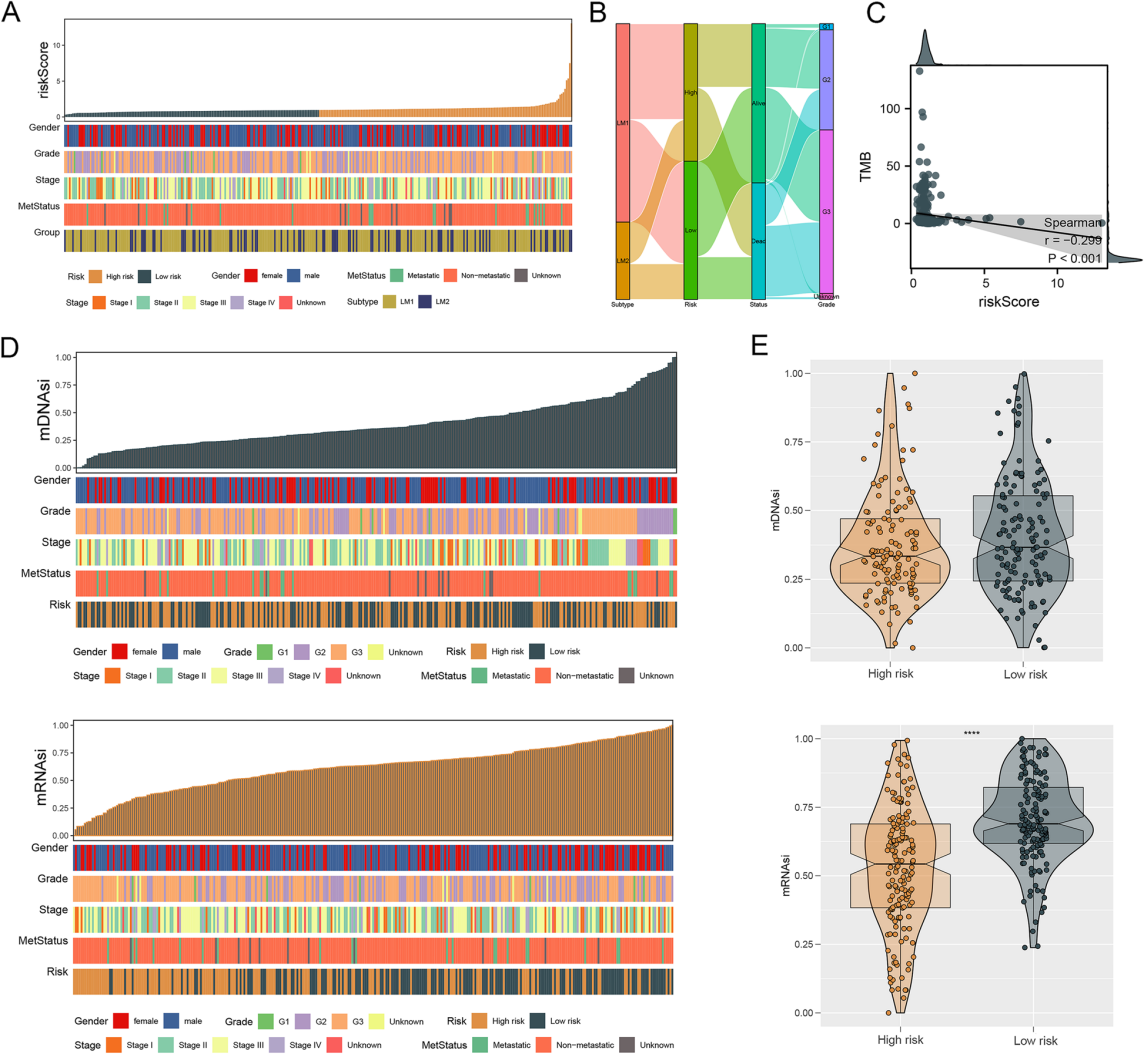
Correlation of LMGs with clinical features, TMB levels, and stemness index. Notes: **A** The patients’ risk scores and clinical information were presented. **B** The association between LM subtypes and risk groups as well as the status and grade was displayed. **C** TMB levels between the two risk groups were compared. **D** The association between the clinical and molecular features (sex, MetStatus, grade, stage, and Risk) and mRNAsi as well as mDNAsi levels. **E** Higher mDNAsi and mRNAsi levels were observed in patients with low risk than those with high risk

The risk score for each patient was presented along with clinical information (Fig. 7A). An alluvial diagram was used to display the association between LM subtypes and risk groups as well as the status and grade of each patient (Fig. 7B). As well, we compared TMB levels in two risk groups. Patients with a lower risk score had higher levels of TMB (Fig. 7C). Additionally, mDNAsi and mRNAsi levels were quantified in patients (Fig. 7D). Patients in the low-hazards group had higher mDNAsi and mRNAsi levels than those in the high-hazards group. (Fig. 7E).

### An analysis of the immune profiles of two risk groups

**Fig. 8:**
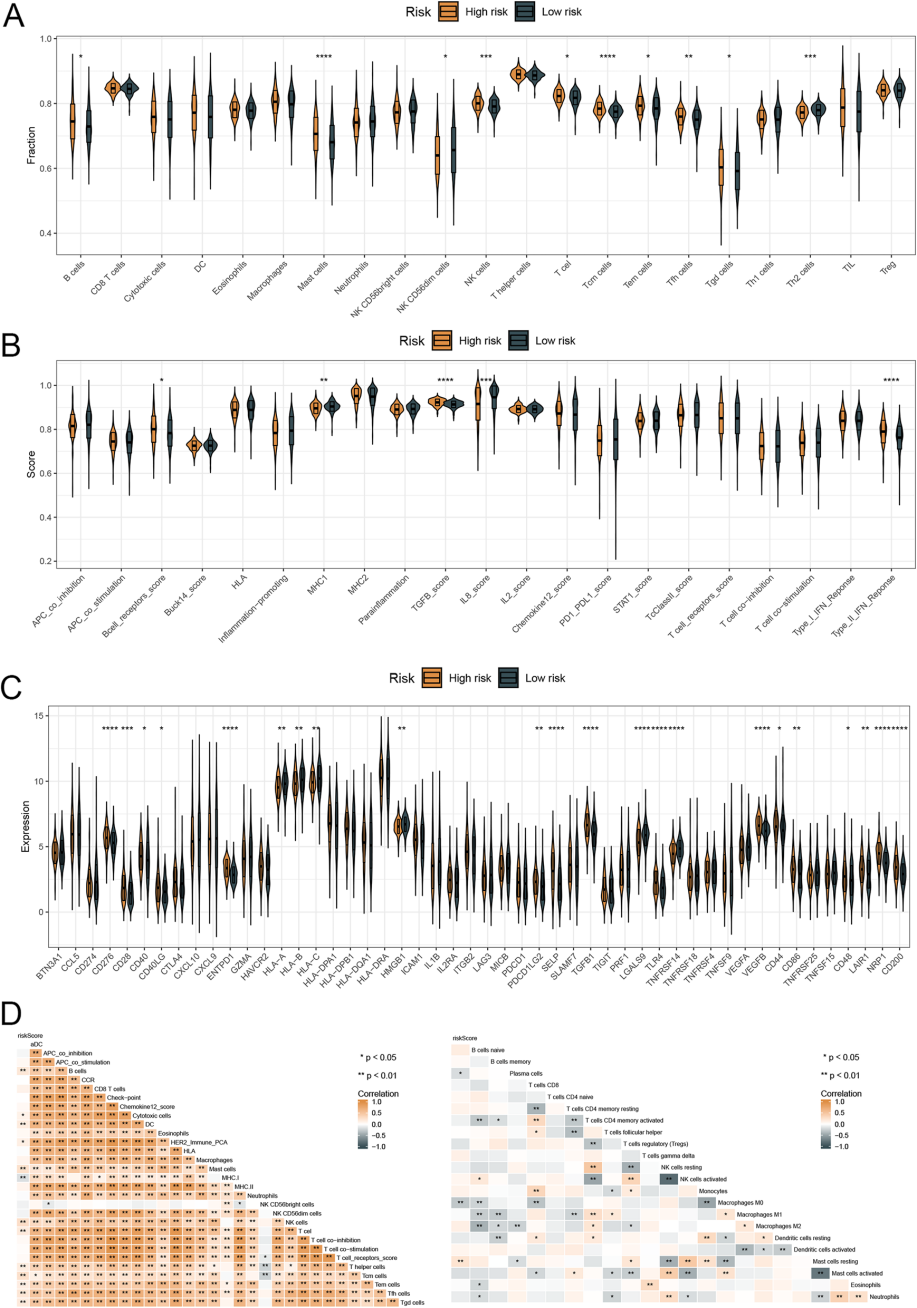
Immune checkpoints’ scores and the scores of immune features between two risk groups. Notes: **A** and **B** Higher levels of immune cells and immune function scores were observed than those in the low risk group. **C** The expression levels of immune checkpoints’ scores were compared between patients with high- and low-risk. **D** The correlation between risk score and the scores of immune features was estimated

**Fig. 9:**
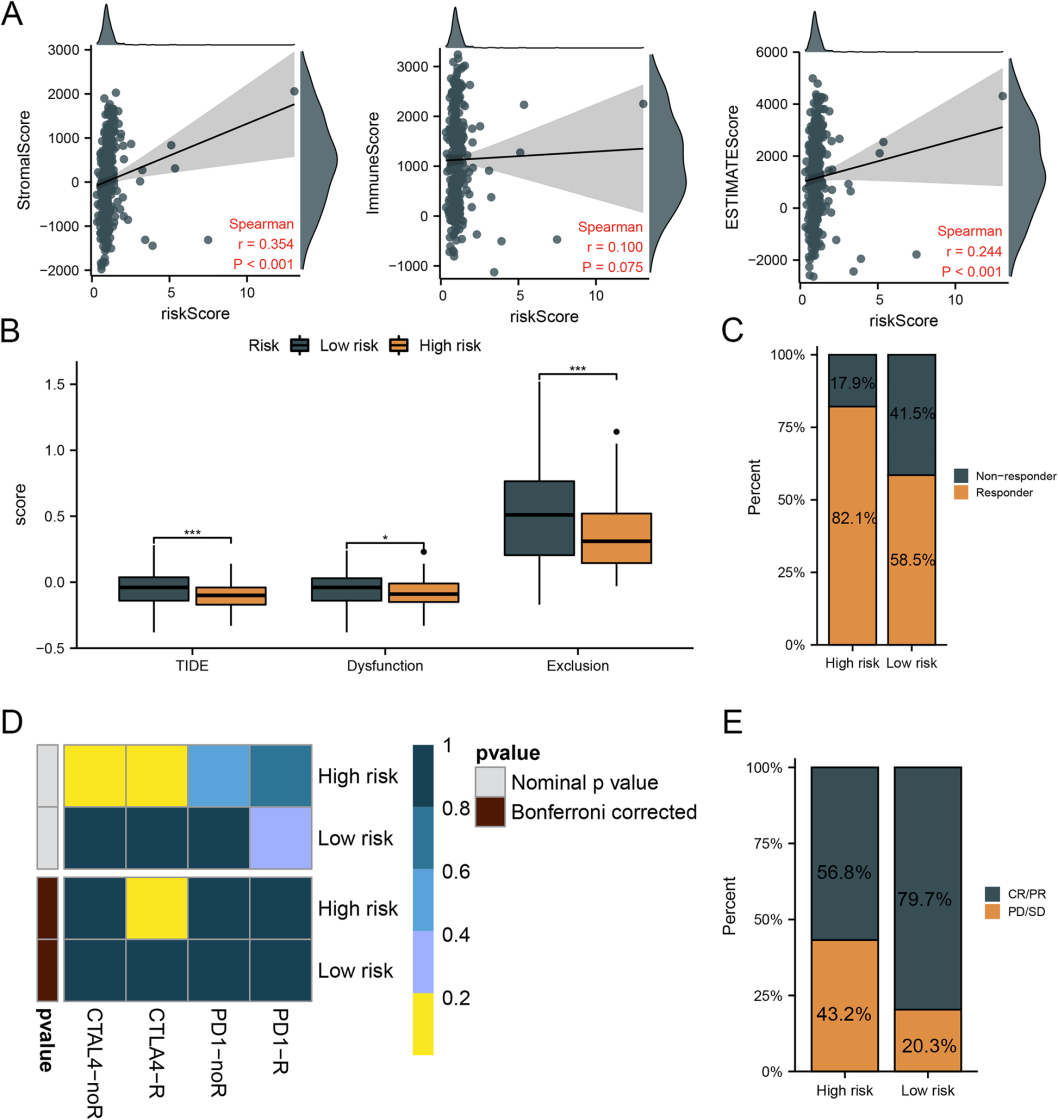
Immunotherapeutic responses in two risk groups. Notes: **A** The correlation between risk score and immune and ESTIMATE scores was estimated. **B** The difference in TIDE, Dysfunction, and Exclusion scores between patients in high and low risk groups was analyzed. **C** A larger percentage of patients in the high-risk group responded to immunotherapy than those in the low-risk group. **D** The immunotherapy targeting CTLA4 receptors was observed to be effective for patients with high hazards. **E** The effectiveness of clinical immunotherapy in patients between two risk groups was compared

Results of ssGSEA showed that patients with high hazards had higher levels of immune cells and immune function scores than those in the low risk group (Fig. 8A, B). Furthermore, patients with high hazards had obviously higher immune checkpoints’ scores than patients in the low-risk group (Fig. 8C). By using heatmaps, we showed the correlation between risk score and the scores of immune features (Fig. 8D). There was a significant positive correlation between risk score and immune and ESTIMATE scores, indicating that the infiltration of immune cells increases with risk score (Fig. 9A). There was a significant difference between patients in high- and low-risk groups for TIDE, Dysfunction, and Exclusion scores (Fig. 9B). A total of 82.1% of patients in the high-hazards group responded to immunotherapy, and 58.5% of patients in the low-hazards group responded (Fig. 9C). Furthermore, subclass mapping results indicated that immunotherapy targeting CTLA4 receptors was effective for patients with high hazards (Fig. 9D). Finally, we compared the effectiveness of clinical immunotherapy in patients in the two risk groups in the TCGA cohort. After receiving immunotherapy, 56.8% of patients with high hazards and 79.7% of patients in the low-risk group experienced complete remissions or partial remissions (Fig. 9E).

### Validation of tumor-promoting effects of CYP19A1 in gastric cancer

**Fig. 10:**
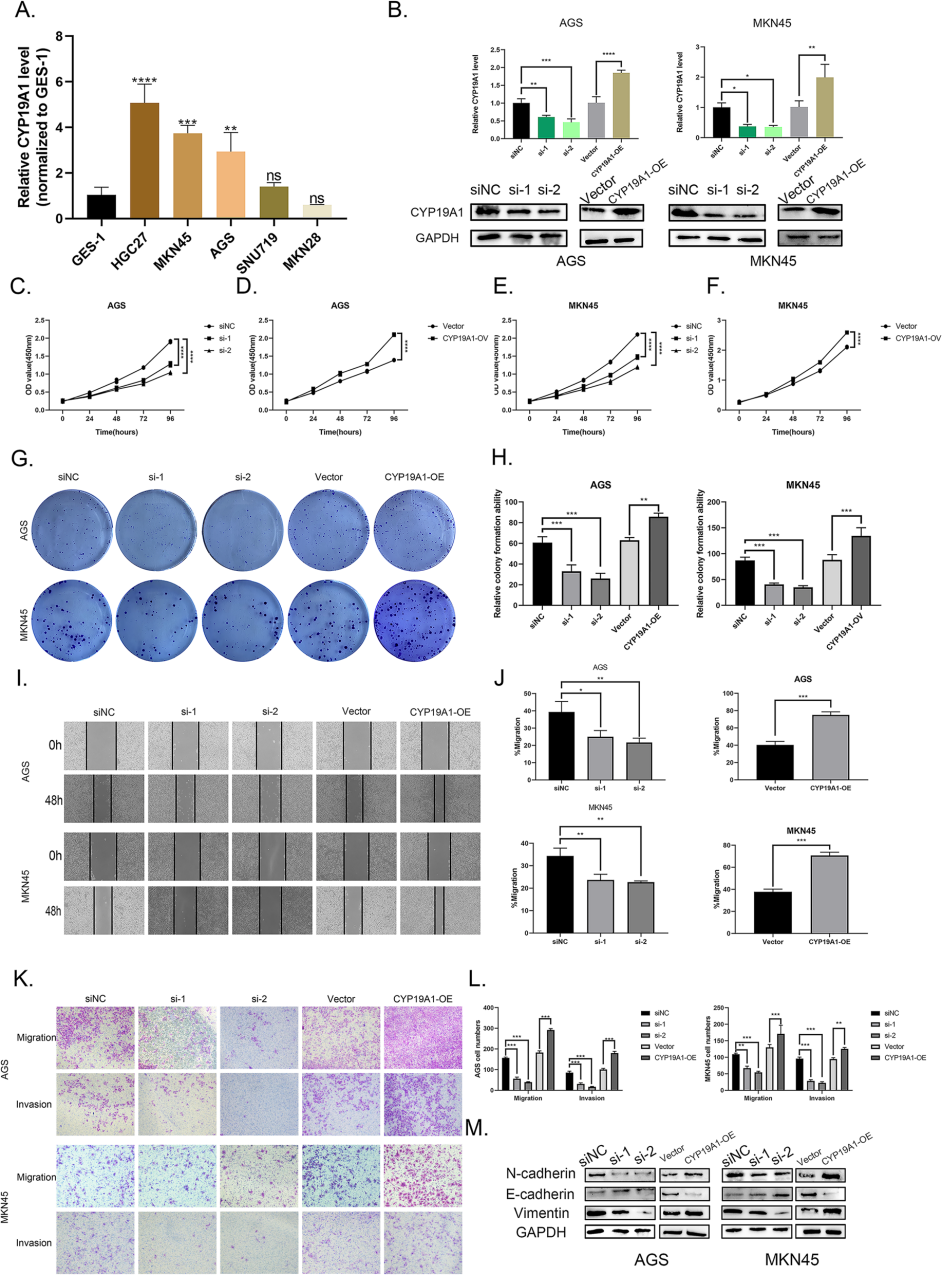
Validation of tumor-promoting effects of CYP19A1 in gastric cancer. Notes: **A** The expression level of CYP19A1 in several gastric cancer cell lines examined by qRT-PCR. **B** Knock-down and over-expression efficiencies of CYP19A1 were determined by qPCR and western blot. **C-F** CCK-8 assay was performed to explore the effect of proliferation of CYP19A1 in AGS and MKN45 cell lines. **G** and **H** The colony formation assay was used to validate the role CYP19A1 plays in the proliferation of AGS and MKN45 cell lines and the colonies were calculated. **I** and **J** The wound healing assay was used to explore the effect of migration of CYP19A1 in AGS and MKN45 cell lines and the rate of migration was calculated. **K** and **L** Transwell assays were used to validate the role CYP19A1 plays in the migration and invasion of AGS and MKN45 cell lines and the number of cells was calculated. **M** We carried out western blots and analyzed the expression of N-cadherin, E-cadherin, and vimentin to determine the correlation between CYP9A1 and EMT. The blots used in this figure conformed to the digital image and integrity policies. The blot and the corresponding internal reference in the same group were cropped from the same membrane and blots from different groups were from different membranes. The membrane was cut during the process of western blots according to the molecular mass of the target protein prior to hybridization with antibodies. One fuller-length, original, unprocessed blot performed with my samples for each antibody which confirms specific detection of the target antigen was provided in the supplementary material

On the basis of the results of differential expression analysis (Fig. 6E) and analysis for risk factors (Fig. 6C), we selected the gene CYP19A1 as our target gene which exhibited the highest relative expression in tumor tissues and demonstrated the most obvious correlation with the worse survival prognosis. Above all, quantitative real-time PCR (qRT-RCR) was used to validate the expression level of CYP19A1 in several common gastric cancer cell lines and the results showed that some of the gastric cancer cell lines exhibited high CYP19A1 expression with statistical significance (Fig. 10A). We selected two cell lines, AGS and MKN45, whose expression levels of CYP19A1 were intermediate to knockdown or over-express CYP19A1. Western blots and qRT-RCR were performed to measure knockdown and over-expression efficiency at both mRNA and protein levels (Fig. 10B). Then, the above-mentioned cell lines verified by us were employed to carry out CCK-8 assay, colony formation assay, wound healing assay, and transwell assay to explore the role of CYP19A1 in the cell proliferation, migration, and invasion and the results were counted and statistically analyzed which demonstrated the ability of CYP19A1 to promote the progression of gastric cancer (Fig. 10C-L). The correlation between epithelial-mesenchymal transition (EMT) and CYP19A1 was also studied by examining the expression of EMT-related proteins consisting of N-cadherin, E-cadherin, and vimentin by western blots and we obtained positive results (Fig. 10M).

## Discussion

GC is the fifth most commonly diagnosed malignancy, with an estimated 26,380 new cases and 11,090 new deaths in 2022 [1, 22]. Heterogeneity is a hallmark of GC. Patients with the same TNM stage may differ from each other in prognoses and respond differently to immunotherapy [23]. Despite the improvement in GC treatment over the last few years, the survival of patients remains unsatisfactory [24, 25]. The effectiveness of GC treatments still needs to be improved in order to prolong patients' 5-year survival. Besides chemotherapy and radiotherapy, targeted therapy will also play a unique role in the treatment of GC [26]. Therefore, there is an urgent need to find new molecular biomarkers of GC at the genetic level.

Lipids, lipid metabolites, and downstream effector molecules form a large signal network. Changing lipid metabolites affects the signaling network, thus promoting cancer progression [6]. The invasion and metastasis of malignancy are facilitated by biologically active lipids such as prostaglandin E2, leukotrienes, sphingosine-1-phosphate, and cholesterol esters [27–29]. Fatty acid synthase, a key enzyme for lipid metabolism, can promote tumor progression in several ways [30]. It is expected that STAD can be effectively treated by targeting genes involved in lipid metabolic pathways.

Using differentially expressed and prognostic-related intersection genes, we used unsupervised clustering to divide STAD patients into the two subtypes. Variance analyses between the two subtypes were performed based on prognosis, clinical data, pathways enrichment, genomic features, immune infiltration, and response to immunotherapy. Prognosis-related genes were used for random forest analysis to screen hub genes and build prognostic models for further evaluation. It was indicated in the findings that the expression of LMGs was strongly related to prognosis, genomic alterations, and immune features. The expression of LMGs was also capable of predicting immunotherapy response well.

Through TCGA cohort analysis, a five-gene signature was identified as a reliable predictor of survival. There was a significant difference in survival cure rates between the two risk groups, demonstrating that the expression level of identified LMGs was closely related to patients’ outcomes. Then the manifestation of this signature was well verified in the GEO cohort. We verified that the respective expressions of the five genes predicted prognosis, and the results were also meaningful. In addition, we examined the genomic alterations and immune features of patients in two risk groups. TMB is an indicator of a patient's response to the ICIs treatment, regardless of PD-L1 expression levels [31, 32]. A relatively negative correlation between TMB levels and risk score was validated. Cancer stem cells may affect the progression, recurrence, and metastasis of cancer [33, 34]. The mDNAsi index and mRNASi index are based on DNA methylation levels and mRNA expression levels, respectively, and reflect epigenetic dry characteristics and transcriptomic dry characteristics [35]. Patients with low hazards had higher mDNAsi and mRNAsi than those in the high risk group.

After that, we performed immune-related analyses to gain a deeper understanding of STAD's immune landscape. The ssGSEA analysis showed that patients with high hazards had higher scores of immune cells, immune function, and immune checkpoints than those in the low-risk group. There was a clear positive correlation between the stromal scores, ESTIMATE scores and risk scores. By targeting PD1, PD-L1 and CTLA4, multidisciplinary treatments for cancer have improved [36]. Consequently, TIDE analysis was used to predict patients’ immunotherapy response. The results showed that more patients with high hazards responded to immunotherapy than those in the low-risk group. This result was validated by the calculation of subclass mapping and clinical immunotherapy efficacy in TCGA patients. Based on these findings, ICIs may be beneficial for patients with high hazards.

Despite some merits, the limitations in our study were also nonneglectable. Firstly, the underlying mechanism of how LMGs affect prognosis in patients with gastric cancer is still unclear. Further experiments are needed to explore their molecular mechanism. Secondly, this model used a GEO dataset for validation, which was not universal and still required more cohorts for validation. Then, as more and more LMGs are excavated, the five genes identified in our study may be incomplete, which means that prognostic characteristics of LMGs in gastric cancer should be updated. In addition, the joint model of objective response rate (ORR) and time to event (TTE) has caught our attention and seems to be able to improve prediction accuracy [37], which could ensure better fault tolerance and provide new ideas for our future studies. Last but not least, only the role CYP19A1 plays in gastric cancer was explored and the molecular mechanisms underlying this phenomenon remain studied.

### Supplementary Information

**Additional file 1: Figure S1.** Original western blots.

## Data Availability

The datasets analyzed during the current study could be found in the TCGA database (https://portal.gdc.cancer.gov/) and the GEO database (https://www.ncbi.nlm.nih.gov/geo/). The authors declared that all data supporting the conclusions of this research is available. Experimental data in this study are available from the corresponding author upon reasonable request.
