# Supplementary material for: CYP19A1 promotes gastric cancer as part of a lipid metabolism-related gene signature related to the response of immunotherapy and prognosis
Source: BMC Med Genomics. 2023 Oct 2;16:228. doi: 10.1186/s12920-023-01664-y (PMC10544546; doi:10.1186/s12920-023-01664-y)
Supplement: Supplementary file 1 — Additional file 1: Figure S1. Original western blots. [file 12920_2023_1664_MOESM1_ESM.pdf]

Original western blots

Figure 10B

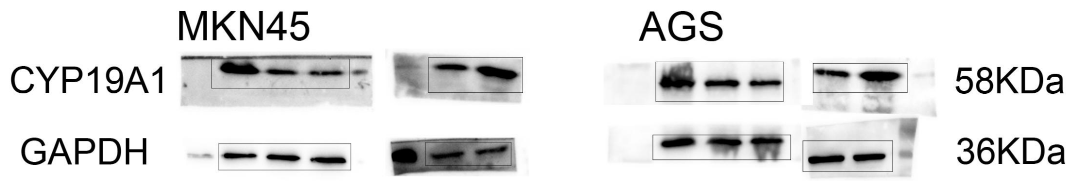

Figure 10M

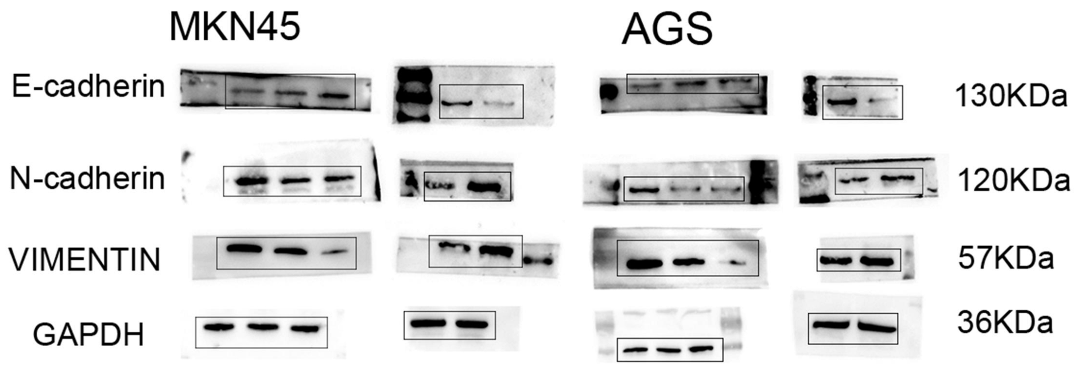

Replicates of western blots

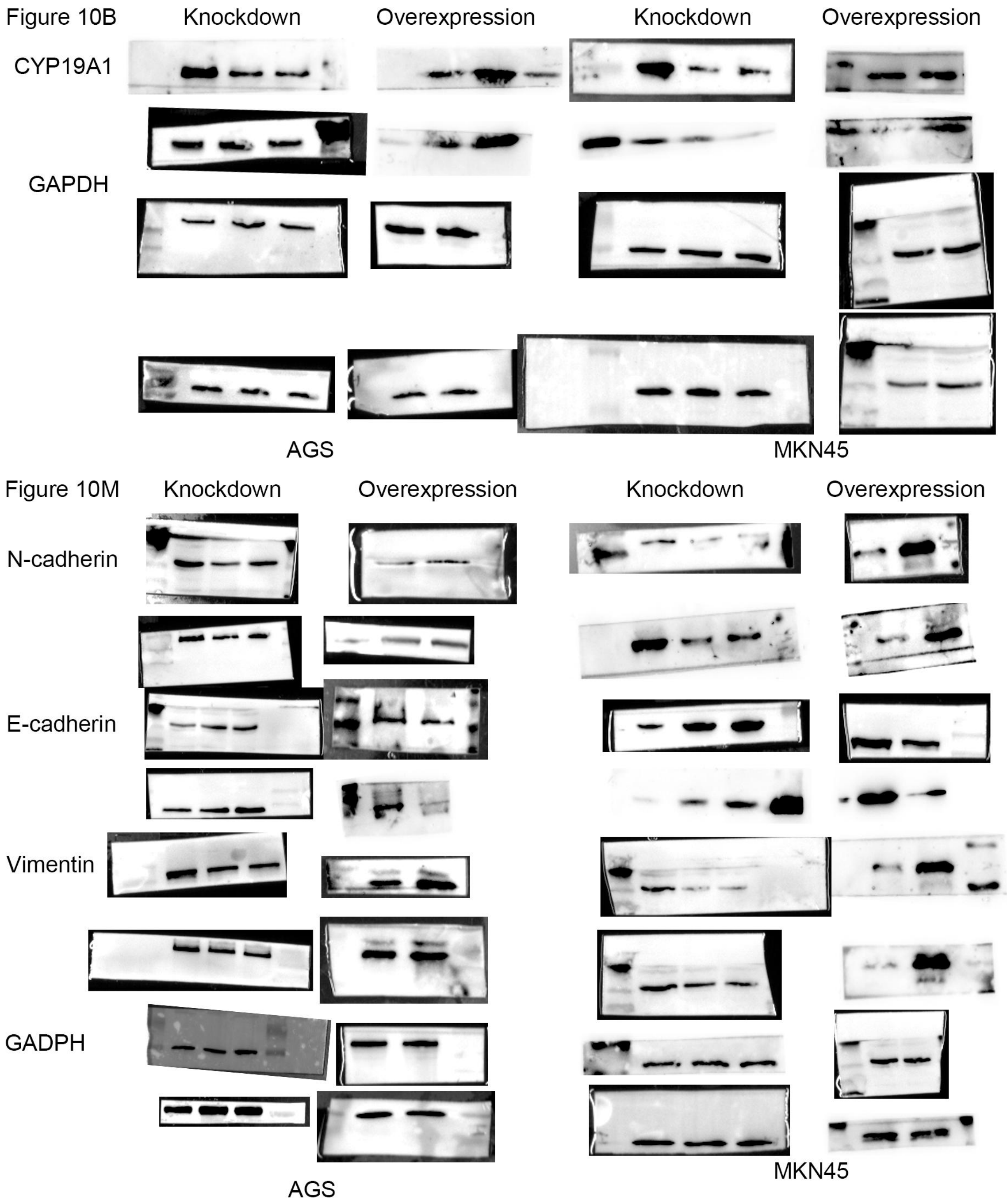

Full-length, original, unprocessed blots performed with your samples for each antibody

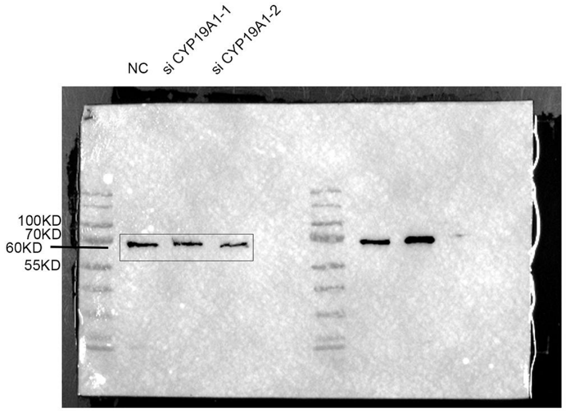

CYP19A1

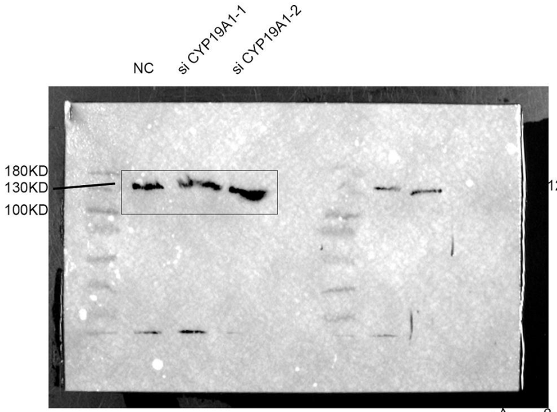

E-cadherin

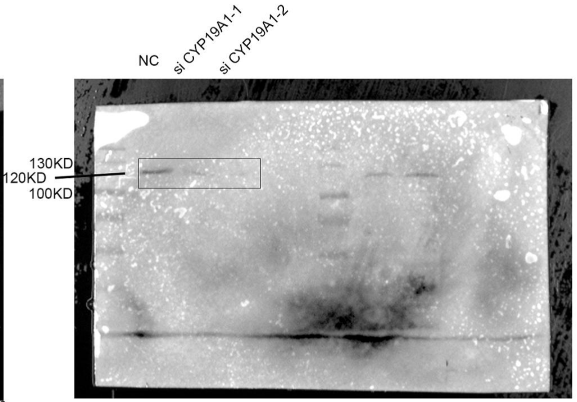

N-cadherin

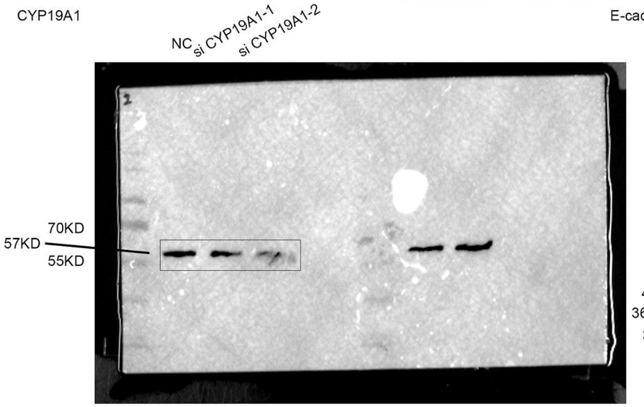

Vimentin

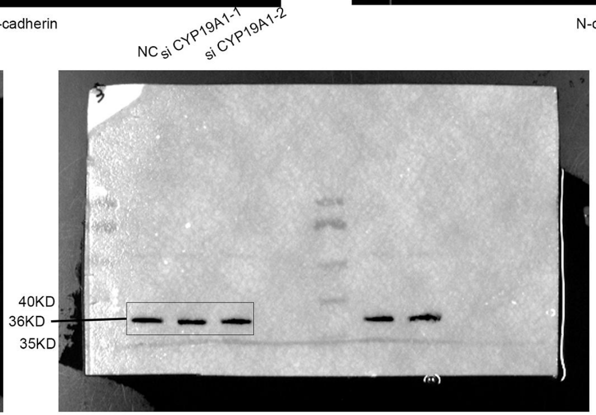

GAPDH
